# Supplementary material for: Analysis of PPARγ Signaling Activity in Psoriasis
Source: Int J Mol Sci. 2021 Aug 10;22(16):8603. doi: 10.3390/ijms22168603 (PMC8395241; doi:10.3390/ijms22168603)
Supplement: Supplementary file 1 [file ijms-22-08603-s001.zip › Supplemental materials_Analysis of PPARg signaling activity in psoriasis/Pathway models/Models images and html files/Model of PPARG related pathways in psoriasis (short version)/286194.html]

Ca2+


# Small Molecule Ca2+

|  |  |
| --- | --- |
| URN | urn:agi-cas:14127-61-8 |
| Total Entities | 0 |
| Connectivity | 19949 |
| Name | Ca2+ |
| Class | Endogenous compound |
| Molecular Weight | 40.078000 |
| ObjectType | Small Molecule |

---

|  |  |
| --- | --- |
| Pathway | Alzheimer's Disease |
|  | Circadian Clock Genes Mutations Cause Insomnia |
|  | Skin Aging |
|  | Apoptosis of Cochlear Hair Cells (Noise-Induced, Mouse Model) |
|  | mTOR Signaling Activation by Fatty Acids and Glucose |
|  | mTOR Signaling Activation by Amino Acids |
|  | mTOR Signaling |
|  | mTOR Signaling Hyperfunction |
|  | Complement Activation by Lectin |
|  | Complement Classical Pathway |
|  | Ion Channels of Stomach in Appetite Regulation |
|  | High Fat Diet Activates cAMP Related Exocytosis in Synapse and Endocrine Cell |
|  | GCG and PPY Regulate Metabolism and Satiety |
|  | Ghrelin and Nesfatin-1 Regulate Stomach Functional State and Satiety |
|  | mTOR Signaling Activation by Taste Sensor Receptors (Rodent Model) |
|  | Ghrelin and SST Regulate GH1 Release in Hypothalamus |
|  | Taste Receptors Activation in Tongue |
|  | GUCY2C Signaling in Apetite Regulation |
|  | S-cell: Secretin Production |
|  | L-cell: GCG, PYY and 5-HT Release |
|  | P-cell Activation |
|  | I-cell: CCK-8 Secretion and Regulation of Eating Behavior |
|  | D-cell: Somatostatin Release |
|  | A-cell (X-like): Ghrelin and Nesfatin-1 Synthesis |
|  | G-cell: Gastrin Secretion |
|  | ECL-cell: CHGA and Histamine Synthesis |
|  | Insulin Secretion |
|  | Insulin Synthesis in beta-Cell |
|  | Insulin Release Inhibition by Somatostatins |
|  | Norepinephrine Neuronal Release |
|  | Dopamine Receptors Signaling |
|  | Serotonin Receptors Signaling |
|  | Catecholamines Secretion from Adrenal Gland |
|  | Thyroid Hormones Common non-Genomic Effects in Hyperthyroidism |
|  | Circadian Clock and Sleep Regulation by Melatonin |
|  | Norepinephrine Release Regulation |
|  | Dopamine Mediated Glutamate Release/Uptake Circle in Neuron in Migraine |
|  | Dopamine Synthesis and Release |
|  | CRH Secretion Regulation |
|  | Prolactin Production |
|  | POMC Secretion in adenohypophysis |
|  | Oxytocin Action in Mammary Gland |
|  | Thrombopoietin Receptors Signaling in Platelet Maturation |
|  | Estrogens in Prolactin Production |
|  | Mineralcorticoids Secretion Regulation |
|  | Dectin-1 (CLEC7A) Signaling |
|  | Dectin-2 (CLEC6A)/Mincle (CLEC4E)/BDCA2 (CLEC4C) Signaling |
|  | T-Cell Dependent B-Cell Activation |
|  | T-Cell Independent B-Cell Activation |
|  | V(D)J Recombination Activation |
|  | Natural Killer Cell Activation through C-Type Lectin-like Receptors |
|  | Natural Killer Cell Activation through ITAM-Containing Receptors |
|  | Natural Killer Cell Activation through ITSM-Containing Receptors |
|  | Natural Killer Cell Activation through Integrins and non-ITAM-Containing Receptors |
|  | T-Cell Maturation (Hypothesis) |
|  | Natural Killer Cell Activation |
|  | Peripheral T-Cell Tolerance |
|  | T-Cell Central Tolerance |
|  | CD8+ T-Cell Activation |
|  | Th1-Cell Differentiation |
|  | Treg-Cell Differentiation |
|  | Th2-Cell Differentiation |
|  | T-Cell Receptor Signaling |
|  | CXC Chemokine Receptor Signaling |
|  | CC Chemokine Receptor Signaling |
|  | Plasmin Effects in Inflammation |
|  | Platelet Activation via Adhesion Molecules |
|  | Scavenger Receptors in Platelet Activation |
|  | Scavenger Receptors in Platelet Aggregation |
|  | Proplatelet Maturation |
|  | Bradykinin Effects in Inflammation |
|  | Platelet Activation via GPCR Signaling |
|  | Coagulation Cascade |
|  | Necrosis |
|  | Lipoxin A4/FPR2-Related Neutrophil Depression |
|  | Annexins in Inflammation Resolution |
|  | Eat me Signal: Apoptotic Cell Initiates Phagocytosis |
|  | Eat me Signal: Apoptotic Cell Induces Phagocytosis |
|  | Leukotriene Effect on Vascular Endothelial Cell Response |
|  | Eicosanoids in Myeloid Cell Activation |
|  | Leukotriene Effect on Airway Smooth Muscle Response |
|  | Histamine Receptor H3 Signaling in Neurons |
|  | Prostaglandin E2 and Histamine in Gastric Acid Secretion |
|  | Basophil Activation |
|  | Eosinophil Activation |
|  | Mast-Cell Activation via IgE Signaling |
|  | Mast-Cells De Novo Synthesized Mediators via IgE Independent Signaling |
|  | CD16/CD14 Proinflammatory Monocyte Activation |
|  | Macrophage M2-Related Phagocytosis |
|  | anti-Inflammatory Function of Macrophage M2 Lineage |
|  | Neutrophil Recruitment and Priming |
|  | Neutrophil Activation via FCGR3B |
|  | Neutrophil Degranulation via FPR1 Signaling |
|  | CR3 Mediated Phagocytosis in Neutrophils and Macrophages |
|  | Neutrophil Chemotaxis |
|  | Neutrophil Activation via Adherence on Endothelial Cells |
|  | SNS Related Adrenal Cortex Response |
|  | Vascular Endothelial Cell Activation by NO |
|  | Vascular Smooth Muscle Cell/Pericyte Migration and Proliferation |
|  | Vasodilation Activation |
|  | Vascular Endothelial Cell Activation by Cytokines |
|  | Vascular Endothelial Cell Activation by Blood Coagulation Factors |
|  | Vascular Endothelial Cell Activation by Growth Factors |
|  | Vascular Endothelial Cell Permeability Activation |
|  | Dendritic Spine Morphogenesis and Stabilization |
|  | Synaptic Potentiation by PKMZ in LTP Maintenance - Active State |
|  | Potassium Flux Regulation |
|  | GFs/TNF -> Ion Channels |
|  | Nociception Expression Targets Signaling |
|  | Sodium Flux Regulation |
|  | Vascular Motility |
|  | Ca2+ Flux Regulation |
|  | TAAR1 -> Neurotransmitter Uptake |
|  | NPY1R -> CRH/POMC Production |
|  | NTSR1 Expression Targets |
|  | CGRP -> Calcium Influx |
|  | CRH -> Synthesis of Corticosteroids |
|  | EDNRA/B -> Vascular Motility |
|  | CCKBR -> Neurotransmitter Uptake |
|  | ADORA1/2A -> Exocytosis |
|  | ADORA3 -> Mast-Cell Degranulation |
|  | ADRA1 -> Vasoconstriction |
|  | ADRA1A -> IL6 Production |
|  | ADRA1 -> Ion Channels |
|  | ADRA2C/ADRB2 -> Vasoconstriction |
|  | ADRA2C/ADRB2 -> Synaptic Endocytosis |
|  | ADRA2A -> Neurotransmitter Release |
|  | ADRA1 -> Prostaglandin Generation |
|  | BDKRB1/2 -> Ion Channels |
|  | BDKRB1/2 -> Prostaglandin Generation |
|  | CNR1/2 -> Membrane Transport |
|  | CNR1/2 -> Vascular Motility |
|  | CNR1/2 -> IL1B/2/4/6/10 Production |
|  | DRD2 Expression Targets |
|  | DRD2 -> TRPC1 Transcription |
|  | DRD2/4 -> Membrane Transport |
|  | GALR1/2/3 -> POMC/NPY Production |
|  | GALR1/2/3 -> Neurotransmitter Metabolism |
|  | HRH1/2 -> Membrane Polarization |
|  | HRH1/3 -> Synaptic Transmission |
|  | HRH1/2 -> Vascular Motility |
|  | HRH2/4 -> IL6/10 Production |
|  | CHRM1 -> IL2 Production |
|  | CHRM1/2/3/5 -> Ion Channels |
|  | CHRM1/2/3 -> Vascular Motility |
|  | OPRK -> Pain Perception |
|  | PTGFR -> Vasoconstriction |
|  | PTGIR -> IL6 Production |
|  | PTGER1/4 -> Vascular Motility |
|  | PTGER2/3 -> Vascular Motility |
|  | PTGER2/3 -> Inflammation-Related Expression Targets |
|  | HTR1 -> IL6 Production |
|  | HTR5 -> TNF Production |
|  | HTR3A -> Cation Transport |
|  | HTR2 -> Membrane Transport |
|  | HTR1 -> Membrane Transport |
|  | HTR1 -> Vascular Motility |
|  | TACR1 -> Membrane Transport |
|  | TACR1 -> TNF/IL6/IL8 Production |
|  | CHRNA3-B4/A4-B2/A7 -> Ion Transport |
|  | CHRNA7 -> NOS1 Production |
|  | GABA(B)R -> Postsynaptic Inhibition |
|  | AMPA Receptors -> Calcium Influx |
|  | NMDA Receptor -> Synaptic Excitation |
|  | GRM1/5 (Postsynaptic) -> Ion Channels |
|  | NMDA Receptors -> Ca2+/CREB Activation/PGE2 Synthesis |
|  | P2RY2/12/13/14 -> N-Type Calcium Channel |
|  | P2RY11/13/14 -> IL8/10 Production |
|  | P2RY1/2/4/6 -> Potassium Channels |
|  | P2RXs -> Synaptic Transmission |
|  | NTRK1/2/3 -> Acetylcholine Production |
|  | EPHB -> NMDA Receptor Activation |
|  | Kidney Cells Cilia Organization |
|  | Luteinizing Hormone in Oocyte Meiotic Resumption |
|  | Agomelatine Antidepressant Action |
|  | Histamine in Arousal Regulation |
|  | Circadian Clock in Sleep Regulation |
|  | Endocannabinoids Role in Sleep Regulation |
|  | Circadian Clock Genes in Suprachiasmatic Nuclei Neurons |
|  | Hypocretin (Orexin) in Arousal Regulation |
|  | Adenosine Role in Sleep Regulation |
|  | Retinal Ganglion and Suprachiasmatic Nucleus in Melatonin Synthesis Regulation |
|  | RHO Signaling (Light Signal Termination and Photoreceptor Recovery) |
|  | RHO Signaling (Light) |
|  | RHO Signaling (Dark) |
|  | non-Genomic Rapid Actions of Vitamin D in Vitamin D Biology |
|  | Apoptosis |
|  | G0/G1 Phase Transition |
|  | Gap Junction Assembly |
|  | Desmosome Assembly |
|  | Adherens Junction Assembly (Cadherins) |
|  | Actin Cytoskeleton |
|  | Actomyosin-Based Movement |
|  | Exocytosis: Vesicle Priming |
|  | Intraflagellar Transport: Retrograde Transport |
|  | Transcytosis |
|  | beta-Cell Granules Exocytois |
|  | Intraflagellar Transport: Anterograde Transport |
|  | Cardiomyocyte Dysfunction |
|  | Th1-Cells Activation and Proliferation in Atherosclerosis |
|  | Dilated Cardiomyopathy |
|  | Alcoholic Dilated Cardiomyopathy (Mouse Model) |
|  | Ca2+ Signaling in Dilated Cardiomyopathy |
|  | Sarcomere in Cardiomyocyte in Dilated Cardiomyopathy |
|  | Cardiomyocyte Hypertrophy |
|  | Hypertrophic Cardiomyopathy |
|  | Sarcomere Disorganization and Intracellular Calcium Overload |
|  | Endothelin Signaling in Arterial Hypertension |
|  | Arterial Hypertension |
|  | Aldosterone Synthesis Acute Regulation |
|  | Endothelial Cell Dysfunction in Arterial Hypertension |
|  | Aldosterone Synthesis Chronic Regulation |
|  | Smooth Muscle Cell Dysfunction in Arterial Hypertension |
|  | TRPM4/6/7/8 Signaling in Arterial Hypertension (Hypothesis) |
|  | Smooth Muscle Cell Dysfunction in Pulmonary Hypertension |
|  | Myocardial Remodeling |
|  | Reperfusion Injury in Myocardial ischemia |
|  | Oxygen Shortage in Myocardial Ischemia |
|  | Myocardial Infarction |
|  | Preconditioning Ischemia |
|  | Vascular Reactivity in Raynaud Disease |
|  | Cold Stress and Adrenoceptor alpha 2C Signaling in Raynaud Disease |
|  | NO Synthesis in Early Vasospasm |
|  | Neurons Necrosis Caused by Energy Deficiency |
|  | Contraction Due Vasospasm |
|  | Atopic Dermatitis Onset |
|  | Mast-Cells Activation in Atopic Dermatitis |
|  | X-Linked Ichthyosis |
|  | Cell Cycle Dysregulation in Melanoma |
|  | WNT Signaling in Melanoma |
|  | Melanoma |
|  | Apoptosis Block in Melanoma Cell |
|  | MITF as a Regulator of Melanoma Cell Development |
|  | Apoptotic Keratinocytes Clearance Recession in Systemic Lupus Erythematosus |
|  | Dendritic Cell Activation in Systemic Lupus Erythematosis |
|  | Th0-Cell Aberrant Activation in Systemic Lupus Erythematosus |
|  | Th1-Cell Function in Systemic Lupus Erythematosus |
|  | B-Cell Activation in Systemic Lupus Erythematosus |
|  | Th2-Cell Function in Systemic Lupus Erythematosus |
|  | Complement System Defects in Systemic Lupus Erythematosis |
|  | Oxidative Stress in Vitiligo |
|  | Acute Cytotoxic CD8+T-Cell Response against Melanocytes |
|  | Vitiligo |
|  | T-Cell Cytotoxic Response against Melanocytes in Vitiligo |
|  | Apoptosis of Melanocyte in Vitiligo |
|  | Humoral Immunity in Vitiligo |
|  | CD8+ T-Cell Response to Self-Determinants in Diabetes |
|  | Treg-Cell Function in Diabetes Mellitus Type 1 |
|  | Natural Killer T-Cell Roles in Diabetes Mellitus Type 1 |
|  | non-Suppressive Treg-Cell in Diabetes Mellitus Type 1 |
|  | TLR2 Signaling in Treg-Cell in Type 1 Diabetes (Animal Model) |
|  | Natural Killer Cell in Diabetes Mellitus Type 1 |
|  | Peripheral Tolerance to Autoantigens Recession in Diabetes Mellitus Type 1 |
|  | Treg-Cell Activation in Diabetes Mellitus |
|  | beta-Cell Death in Diabetes Mellitus Type 2 |
|  | Neonatal Diabetes Mellitus |
|  | Wolfram Syndrome Progression (Hypothesis) |
|  | MELAS Syndrome |
|  | Proliferative Diabetic Retinopathy |
|  | Adiponectin in Insulin Resistance Prevention |
|  | Insulin Resistance in Myocytes Induced by Obesity |
|  | beta-Cell Destruction through Cytokines in Diabetes Mellitus |
|  | beta-Cell Mass Regulation |
|  | beta-Cell Dysfunction by Steroids in Diabetes Mellitus (Hypothesis) |
|  | beta-Cell Recovery in Diabetes Mellitus |
|  | G-Proteins Signaling in Insulin Secretion in beta-Cell |
|  | Nitric Oxide Effects on beta-Cell |
|  | Hyperglycemia and Hyperlipidemia Trigger beta-Cell Apoptosis |
|  | PTPRN/STAT5 in Insulin Synthesis in beta-Cell (Rodent Model) |
|  | beta-Cell Function Inhibition by Ciclosporine and Tacrolimus (Rodent Model) |
|  | Olanzapine Effect on Insulin Secretion in Diabetes Mellitus |
|  | Ghrelin Influence on Insulin Secretion (Hypothesis) |
|  | beta-Cell Function Inhibition by Rapamycin (Rodent Model) |
|  | Hyperparathyroidism, Neonatal Severe |
|  | Ca2+ Reabsorption Decline in Intestine |
|  | Ca2+/Pi Reabsorption Decline in Kidney in Hyperparathyroidism |
|  | DRD2 Pharmacological Inhibition in Hyperprolactinemia |
|  | Ethanol Induced Efects in Hyperprolactinemia |
|  | Bone Remodeling in Hyperthyroidism |
|  | Cardiovascular Effects in Hyperthyroidism |
|  | Thyroid Dysfunction |
|  | Immune System Activation in Hyperthyroidism |
|  | GNRH Release Impairment in Hypogonadotrophic Hypogonadism |
|  | GNRH Signaling Impairment in Hypogonadotrophic Hypogonadism |
|  | KISS1 Production Impairment in Hypogonadotropic hypogonadism |
|  | Myocyte Dysfunction in Hypoparathyroidism |
|  | Hypoparathyroidism, Primary |
|  | Ca2+/Pi Reabsorption Decline in Kidney in Hypoparathyroidism |
|  | Hypothyroidism, Secondary (Central) |
|  | Thyroid Stimulating Hormone (TSH) Resistance in Congenital Hypothyroidism |
|  | Overt Hypothyroidism, Primary |
|  | Thyroid Stimulating Hormone (TSH) Secretion in Overt Hypothyroidism |
|  | Central Hypothyroidism |
|  | Tertiary Hypothyroidism |
|  | Extraocular Muscles Weakness in Myasthenia Gravis |
|  | Prolactinoma Overiew |
|  | CD4+T-Cell Response in Celiac Disease |
|  | CD8+ T-Cell Response in Celiac Disease |
|  | Metastatic Colorectal Cancer |
|  | B-Cell Activation in Crohn's Disease |
|  | OipA Signaling in Helicobacter Pylori Infection |
|  | CagA Phosphorylation Dependent Signaling |
|  | EDN, NRG, NRTN, and GDNF/RET Signaling in Hirschsprung Disease |
|  | Th2-Cell Function in Ulcerative Colitis |
|  | T-Cell Cytotoxic Response in Ulcerative Colitis |
|  | Antiphospholipid Antibodies in Platelets and Coagulation Cascade |
|  | B-Cell Chronic Lymphocytic Leukemia |
|  | Diffuse Large-B-Cell Lymphoma |
|  | Diffuse Large-B-Cell Lymphoma ABC Subtype |
|  | Follicular Lymphoma |
|  | Hodgkin Lymphoma |
|  | Mantle Cell Lymphoma |
|  | mTOR/NF-kB/BCR Signaling Disregulation in Mantle Cell Lymphoma |
|  | Mucosa-Associated Lymphoid Tissue (MALT) Lymphoma |
|  | Splenic Marginal Zone Lymphoma |
|  | Osteoclast Activation in Multiple Myeloma |
|  | Multiple Myeloma |
|  | Platelet and Coagulation Cascade Activation by Antiphospholipid Antibodies |
|  | Hereditary Thrombophilia and Pregnancy Complications |
|  | T-Cell Acute Lymphoblastic Leukemia |
|  | Thrombophilia Hereditary |
|  | TRPM7/8 in Pancreatic Adenocarcinoma |
|  | Eosinophil Survival in Asthma |
|  | Eosinophil Activation and Degranulation in Asthma |
|  | Airway Smooth Muscle Cell High Rate Proliferation |
|  | IgE Induces Airway Smooth Muscle Cell Proliferation |
|  | Airway Smooth Muscle Cell Contraction |
|  | Goblet-Cell Exocytosis in Asthma |
|  | Goblet-Cell Related Mucus Secretion in Asthma |
|  | Neutrophil Degranulation via FPR1/IL8 |
|  | Basophil Activation in Asthma |
|  | Mast-Cell Activation in Asthma |
|  | Mast-Cell Related Chemokines Signaling in Asthma |
|  | Antigen-Presenting Cell Role in Asthma |
|  | Th2-Cell Response in Asthma |
|  | CFTR Facilitates Oxidative Stress in Airway Epithelium in CF |
|  | Airway Epithelial Cell Dysfunction |
|  | CFTR in Sperm Capacitation and Acrosome Reaction |
|  | CFTR Expression in Epithelial Cells (Class I Mutations) |
|  | CFTR Related Ion-Channel Dysfunction in Cystic Fibrosis Airway Epithelium (Class III Mutations) |
|  | Osteoclast Activation in Gout |
|  | Steroidogenesis Impairement in Polycystic Ovary Syndrome |
|  | Ovulation Block |
|  | Polycystic Ovary Syndrome |
|  | FSH Action in Polycystic Ovary Syndrome |
|  | PTPN22 Role in Rheumatoid Arthritis |
|  | Osteoclast Activation in Rheumatoid Arthritis |
|  | B-Cells Function in Systemic Scleroderma |
|  | Th2-Cells Function in Systemic Scleroderma |
|  | WHIM Syndrome |
|  | Dentin Formation Disruption |
|  | CCR5 Signaling in Macrophages |
|  | CD8+ T-Cell Action Impairment in HIV Type 1 Infection |
|  | CD4+ T-Cell Function Decline in HIV |
|  | Acinar Cells in the Saliva Formation |
|  | Epithelial Cell in the Saliva Formation |
|  | TNF Receptor Associated Periodic Syndrome |
|  | Glutamate Toxiicity Effects on Striatum Medium Spiny Neurons in Hantington's Disease |
|  | ER Stress in Huntington Disease |
|  | Rod Photoreceptor Signaling Impairment in Retinitis Pigmentosa |
|  | Proteins Mutated in Cochlear Hair Cell Synapse |
|  | Rett Syndrome |
|  | Timothy Syndrome |
|  | IL6 Overexpression After Contraction in Skeletal Muscles in Muscular Dystrophies |
|  | Ca2+ Overload in Muscular Dystrophies |
|  | Muscular Dystrophies |
|  | Dystrophin Glycoprotein Complex Signaling in Muscular Dystrophies |
|  | IGF1 Role in Muscle Hypertrophy |
|  | Myostatin-IGF1 Crosstalk in Skeletal Muscles in Muscular Dystrophies |
|  | Bone Mineralization Impairement in Osteoporosis |
|  | Ca2+ Reabsorption Decline in Kidney |
|  | Hyperparathyroidism, Secondary Effect |
|  | Ca2+ Absorption Decline in Intestine in Osteoporosis |
|  | Bone Resorption by Osteoclasts in Osteoporosis |
|  | Mitochondrial Dysfunction in Alzheimer's Disease |
|  | APP and Glutamate Signaling Cause Neuronal Dysfunction in Alzheimer Disease |
|  | Mitochondrial Respiratory Chain Dysfunction |
|  | Tricarboxylic Acid Cycle Enzymes Involvement in Alzheimer Disease |
|  | Amyloid beta Traffic and Degradation in Extracellular Matrix in Alzheimer's Disease |
|  | Mitochondria Enlargement and Apoptosis in Alzheimer's Disease |
|  | Neurofibrillary Tangle Formation |
|  | Ca2+ Toxicity in Neurons |
|  | ADK Expression Upregulation |
|  | Inherited Channelopathies |
|  | BDNF Upregulation Triggered by Seizures |
|  | Astrocyte Dysfunction and GABA Signaling Deficiency |
|  | Ketogenic Diet Mechanism (Hypothesis) |
|  | Blood-Brain Barrier Disruption in Epileptiform Disorders |
|  | mTOR Hyperactivation after Status Epilepticus |
|  | Glutamate, D-Serine, and ATP Release from Astrocytes |
|  | HMGB1 and IL1B in Neuronal Hyperexcitation |
|  | Apoptosis and Immediate Early Gene Activation in Epileptiform Disorders |
|  | Synaptic Exocytosis in Dementia |
|  | Glioblastoma, Primary |
|  | Glioblastoma, Secondary |
|  | Astrocytoma |
|  | Vascular Endothelial Cell Vasodilation in Brain |
|  | CCL2 Role in CGRP Expression and Release |
|  | Nitroxyl and CGRP in Migraine Pain Activation |
|  | Dopamine Action in CSD Progression |
|  | Dopamine Synthesis and Release in Migraine |
|  | Dopamine Mediated Glutamate Release and Glutamate Uptake Circle |
|  | Vascular Smooth Muscle Cell Vasodilation in Brain |
|  | Postsynaptic Neuron Activation |
|  | Glutamate Overdose and Aura Effect in Familial Hemiplegic Migraine Type 3 |
|  | Glutamate Overdose and Aura Effect in Migraine Type 1 |
|  | eNOS (NOS3) Activation via Prostaglandin E2 and Histamine |
|  | nNOS (NOS1) and iNOS (NOS2) Activation via Glutamate |
|  | Glutamate Overdose and Aura Effect Caused by Mutations in SLC1A3 Gene |
|  | Glutamate Overdose and Aura Effect in Migraine Type 2 |
|  | Prostaglandin E2 Synthesis Activation via Glutamate in Familial Hemiplegic Migraine |
|  | Glutamate Overdose and Aura Effect Caused by Mutations in PRRT2 Gene |
|  | Glutamate Overdose and Aura Effect Overiew |
|  | NO Effect on Vasodilation and Vasoconstriction |
|  | Glutamate Role in Parkinson's Disease |
|  | Glutamate Mediated Excitotoxicity in Amyotrophic Lateral Sclerosis |
|  | MPB-Related Complement Cascade Activation |
|  | SPP1 Induced Survival of Myelin Specific T-Cells in Multiple Sclerosis |
|  | GABRB3 Mutation and Adenosine Deaminase Polymorphism |
|  | Hypocretin Elevation and Monoamine Oxidase Decrease/Inhibition |
|  | Brain Cell Necrosis in Vascular Dementia |
|  | Angiogenesis in Endometriosis |
|  | Coagulation Factors and PAR1/2 Receptors (F2R/F2RL1) in Endometriosis |
|  | EGFR and PTAFR Signaling in Ovarian Cancer |
|  | HPV E5 Regulates EDNRA and EGFR Signaling Pathway in Keratinocytes |
|  | Prostate Cancer |
|  | TRPM8 Effects in Prostate Cancer (Hypothesis) |
|  | Netrin-1 Signaling in Age-Related Macular Degeneration |
|  | Oxidative Stress, All-Trans-Retinal and Lipofuscin Toxicity in AMD |
|  | Ca2+ Toxicity in Lens Cells |
|  | Age-Related Cataract |
|  | VEGFA in Neovascular Glaucoma |
|  | Glutamate Mediated Excitotoxicity in Glaucoma |
|  | PGF2-alpha Effect on Treatment |
|  | Serotonin Signaling in the Ciliary Body |
|  | Potassium Cycling in the Inner Ear (Age-Related) |
|  | Cochlear Hair Cell Synapse Proteins Mutations (Age-Related/Congenital) |
|  | Potassium Cycling in the Inner Ear (Congenital Deafness) |
|  | Potassium Cycling in the Inner Ear (Noise-Induced) |
|  | Cytokines Trigger Otitis Media |
|  | Alveolar Macrophages Dysfunction in COPD |
|  | Muscarinic Receptor-Mediated Airway Smooth Muscle Contraction |
|  | VEGFA Low Expression Level in Chronic Bronchitis |
|  | VEGFA High Expression Level in Pulmonary Emphysema |
|  | Alveolar Epithelial Cell Dysfunction in COPD |
|  | Polycystins Mutation Effects in Cystic Kidney Disease |
|  | Cilia Disorganization |
|  | Penile Detumescense Augmentation Mechanisms |
|  | Penile Erection Impairment |
|  | Endothelial Cell Dysfunction in Glomerulonephritis |
|  | Sperm Motility Impairement in Testicular Male Infertility |
|  | Sertoli Cells Dysfunction Causes Male Infertility |
|  | Hyperammonemia Type I |
|  | Citrullinemia Type I |
|  | Argininemia |
|  | Ammonia Effects on Brain Cells |
|  | Citrullinemia Type II |
|  | Argininosuccinic Aciduria in Urea Cycle Disorders |
|  | Hyperammonemia Type II |
|  | Ions Reabsorption Dysregulation in Nephrolithiasis |
|  | Ca2+ Reabsorption Dysregulation in Urolithiasis |
|  | CD38/CD3 -> JUN/FOS/NF-kB Signaling in T-cell Proliferation |
|  | CD38/CD19 -> JUN/FOS/NF-kB Signaling in B-cell Proliferation |
|  | CD38 -> cADPR/Calcium Signaling |
|  | CD157 -> cADPR/Calcium Signaling |
|  | ANGPT1 -> CREB/CREBBP Expression Targets |
|  | ANGPT2 -> AP-1/CREBBP/MYC Expression Targets |
|  | AREG -> NFATC Expression Target |
|  | AREG -> CREB/CREBBP Expression Targets |
|  | AREG -> NCOR2 Expression Targets |
|  | AREG -> SMAD1 Expression Target |
|  | AREG -> HIF1A Expression Targets |
|  | AREG -> AP-1 Expression Targets |
|  | Noradrenaline/Gs Expression Targets |
|  | Noreadrenaline/Gq Expression Targets |
|  | Epinephrine/Gs Expression Targets |
|  | Acetylcholine Expression Targets |
|  | Serotonin/Gq Expression Targets |
|  | Epinephrine/Gq Expression Targets |
|  | Dopamine/Gi Expression Targets |
|  | B-Cell Receptor -> NFATC Signaling |
|  | B-Cell Receptor -> AP-1 Signaling |
|  | B-Cell Receptor -> NF-kB Signaling |
|  | BTC -> NFATC Expression Targets |
|  | BTC -> AP-1/ATF/CREB Expression Targets |
|  | BTC -> EP300/ETS/ETV/SP1 Expression Targets |
|  | SELE -> ELK-SRF Signaling |
|  | CD2 Expression Targets |
|  | CD19 Expression Targets |
|  | CD81 -> AP-1 Expression Targets |
|  | CD247 Expression Targets |
|  | gamma Globulins Expression Targets |
|  | FCGR3A Expression Targets |
|  | CD19/CR2 Expression Targets |
|  | CD8 -> AP-1 Expression Targets |
|  | CD72 -> CREB/CREBBP Expression Targets |
|  | CD72 -> NF-kB Expression Targets |
|  | CD8 -> ATF/CREB/CREBBP Expression Targets |
|  | CD8 -> NF-kB Expression Targets |
|  | CD80 -> AP-1 Expression Targets |
|  | CD81 Expression Targets |
|  | CD80 -> NF-kB Expression Targets |
|  | CD80 -> NFATC Expression Targets |
|  | CD86 -> NFATC Expression Targets |
|  | CD86-> NFATC Expression Targets |
|  | CD86 -> NF-kB Expression Targets |
|  | CXCL6 Expression Targets |
|  | CXCL1 Expression Targets |
|  | CXCL2 Expression Targets |
|  | CXCL3 Expression Targets |
|  | CXCL5 Expression Targets |
|  | CHRNA7 -> CREB Signaling |
|  | Complement Component GPCRs Signaling |
|  | Acetaminophen Induced Hepatotoxicity |
|  | Dexamethasone Induced Osteoporosis |
|  | Doxorubicin Induced Cardiotoxicity |
|  | Clozapine Induced Granulocytopenia |
|  | Cyclosporine Induced Nephrotoxicity |
|  | EGF -> HIF1A Expression Targets |
|  | EGF -> AP-1/ATF Expression Targets |
|  | EGF -> CREB/CREBBP/ELK/SRF/MYC Expression Targets |
|  | EGF -> MEF/MYOD/NFATC Expression Targets |
|  | EGF -> TP53 Expression Targets |
|  | EGF -> NCOR2 Expression Target |
|  | EGFR -> NCOR2 Signaling |
|  | EGFR/ERBB3 -> MEF/MYOD/NFATC/MYOG Signaling |
|  | EGFR/ERBB2 -> TP53 Signaling |
|  | ERBB2/3 -> EP300/ETS/ETV/SP1 Signaling |
|  | EGFR -> SMAD1 Signaling |
|  | EGFR/ERBB2 -> HIF1A Signaling |
|  | EGFR -> AP-1/ATF2 Signaling |
|  | EGFR -> AP-1/CREB/ELK/SRF/MYC Signaling |
|  | EREG -> CREB Expression Target |
|  | EREG -> EP300/SP1 Expression Targets |
|  | EREG -> HIF1A Expression Target |
|  | EREG -> AP-1/ATF Expression Targets |
|  | Erythropoietin -> AP-1/MYC/CREB Expression Targets |
|  | Erythropoietin -> ELK/SRF Expression Targets |
|  | PECAM1 Expression Targets |
|  | F2 -> AP-1/CREB/ELK/SRF/SP1 Expression Targets |
|  | PLG -> AP-1/CREB/ELK/SRF/SP1 Expression Targets |
|  | FGF9 -> AP-1/CREB/MYC Expression Targets |
|  | FGF10 -> AP-1/CREB/CREBBP/MYC Expression Targets |
|  | FGF1 -> AP-1/CREB/ELK/SRF/MYC Expression Targets |
|  | FGF1 -> NCOR2 Expression Target |
|  | FGF7 -> RUNX Expression Targets |
|  | FGF8 -> AP-1/CREB/MYC Expression Targets |
|  | FGF1 -> RUNX Expression Targets |
|  | FGF18 -> AP-1/CREB Expression Targets |
|  | FGF23 -> NCOR2 Expression Targets |
|  | FGF8 -> RUNX Expression Targets |
|  | FGF9 -> RUNX Expression Targets |
|  | FGF2 -> NCOR2 Expression Targets |
|  | FGF4 -> AP-1/MYC Expression Targets |
|  | FGF7 -> AP-1/CREB/CREBBP/MYC Expression Targets |
|  | FGF2 -> RUNX Expression Targets |
|  | FGF2 -> AP-1/CREB/CREBBP/ELK/SRF/MYC Expression Targets |
|  | FGFR -> RUNX2 Signaling |
|  | FGFR -> AP-1/CREB/CREBBP/ELK/SRF/MYC Signaling |
|  | FPR1 -> Cytoskeleton Signaling |
|  | ADCYAP1 Expression Targets |
|  | FSHR Expression Targets |
|  | Glutamate/Gq Expression Targets |
|  | VIP Expression Targets |
|  | HBEGF -> AP-1/ATF Expression Targets |
|  | HBEGF -> TP53 Expression Targets |
|  | HBEGF -> EP300/ETS/ETV/SP1 Expression Targets |
|  | HBEGF -> HIF1A Expression Targets |
|  | HBEGF -> MEF/MYOD Expression Target |
|  | HBEGF -> CREB/MYC Expression Targets |
|  | HGF -> AP-1/CREB/ELK/SRF/MYC Expression Targets |
|  | HGFR -> AP-1/CREB/MYC Signaling |
|  | EGFR Transactivation in Cancer and Non-Cancer Cells |
|  | TAM Receptor Signaling Activation in Cancer |
|  | G0/G1 Cell Cycle Phase Transition Activation in Cancer |
|  | NFKB Canonical Signaling Activation in Cancer |
|  | Suppression of Caspase Activity in Cancer |
|  | Apoptosis Evasion in Cancer: Overview |
|  | VEGF Independent Angiogenesis in Cancer |
|  | CDH2 Activation Promotes Cancer Cell Migration and Survival |
|  | Invadopodia Formation in Cancer Cells |
|  | Skeletal Muscle Wasting in Cancer Cachexia |
|  | Treg Cells Promote Immunosuppression in Cancer Immune Escape |
|  | Effector T-cell Inactivation in Cancer Immune Escape |
|  | CD2 -> NFATC1 Signaling |
|  | CD19 -> JUN/ELK Signaling |
|  | FcIgER -> NFATC1 Signaling |
|  | FcIgER -> ELK-SRF Signaling |
|  | ICAM1 -> AP-1/CREB/ELK/SRF Signaling |
|  | FibronectinR -> AP-1/ELK/SRF/SREBF Signaling |
|  | CTGF -> AP-1/CREB/MYC Expression Targets |
|  | CTGF -> NCOR2 Expression Target |
|  | ICAM1 Expression Targets |
|  | IL16 -> AP-1 Expression Targets |
|  | IL16-> ATF/CREB/CREBBP Expression Target |
|  | UrokinaseR -> ELK/SRF Signaling |
|  | PTPRC -> BCL6 Expression Targets |
|  | Prostaglandin F Expression Targets |
|  | Thromboxane A2 Expression Targets |
|  | LPA Expression Targets |
|  | PAF/Gq -> AP-1/ATF1/CREB/ERK/SRF Expression Targets |
|  | Dronabinol/Anandamide Expression Targets |
|  | Morphine Expression Targets |
|  | PAF/Gq -> NF-kB Expression Targets |
|  | S1P Expression Targets |
|  | PGE1 Expression Targets |
|  | NRG1 -> MEF/MYOD Expression Targets |
|  | NRG1 -> EP300/ETS/ETV/SP1 Expression Targets |
|  | NRG1 -> CREB/CREBBP/ELK/SRF/MYC Expression Targets |
|  | NRG1 -> TP53 Expression Target |
|  | NRG1 -> AP-1/ATF Expression Targets |
|  | NRG1 -> HIF1A Expression Target |
|  | NGF -> SMAD3/NF-kB Expression Targets |
|  | NGF -> AP-1/TP53/MYC Expression Targets |
|  | NGF -> FOXO/MYCN/ELK-SRF Expression Targets |
|  | BDNF Expression Targets |
|  | NTF3 Expression Targets |
|  | NTF4 Expression Targets |
|  | NGF -> CREB/CEBPB/MEF2A Expression Targets |
|  | Adenosine Expression Targets |
|  | PDGFB -> AP-1/CREB/MYC Expression Target |
|  | PDGFC -> CREB Expression Target |
|  | PDGFD -> AP-1 Expression Targets |
|  | PDGF -> AP-1/CREB/CREBBP/MYC Expression Targets |
|  | KIT -> MITF Signaling |
|  | PDGFR -> AP-1/MYC Signaling |
|  | AVP/Gs -> CREB/ELK/SRF/AP-1/EGR Expression Targets |
|  | GNRH1 Expression Targets |
|  | NTS Expression Targets |
|  | GAST Expression Targets in GAST Expression Targets |
|  | AGT -> TP53 Expression Targets |
|  | GNRH2 Expression Targets |
|  | OXT Expression Targets |
|  | POMC Expression Targets |
|  | CCL11 Expression Targets |
|  | EDN1 Expression Targets |
|  | AGT -> CREB Expression Targets |
|  | AVP/Gs -> MEF/MYOD/NFATC/MYOG Expression Targets |
|  | NPY Expression Targets |
|  | AVP/Gq -> STAT Expression Targets |
|  | IFNA1/Gq Expression Targets |
|  | AGT -> ELK/SRF Expression Targets |
|  | TAC1 Expression Targets |
|  | AVP/Gq -> MEF/MYOD/NFATC/MYOG Expression Targets |
|  | EDN3 Expression Targets |
|  | AVP/Gq -> CREB/ELK/SRF/AP-1/EGR Expression Targets |
|  | PDCD1 -> ATF/CREB/CREBBP Expression Targets |
|  | PDCD1 -> NFATC Expression Targets |
|  | PDCD1 -> AP-1 Expression Targets |
|  | PTPRC -> BCL6 Signaling |
|  | AngiopoietinR -> AP-1 Signaling |
|  | GDNF -> HSF1 Signaling |
|  | NTRK -> AP-1/CREB/ELK/SRF/MYC/SMAD3/TP53 Signaling |
|  | GAS6 -> AP-1/CREB Expression Targets |
|  | FIGF -> AP-1 Expression Target |
|  | FIGF -> NCOR2 Expression Target |
|  | KITLG -> AP-1/CREB/CREBBP/MYC Expression Targets |
|  | GDNF -> HSF1 Expression Targets |
|  | CSF1 -> AP-1/CREB/CREBBP/MYC Expression Targets |
|  | PGF -> AP-1/CREB/CREBBP/MYC Expression Targets |
|  | FLT3LG -> AP-1/CREB/CREBBP Expression Targets |
|  | WNT/Calcium non-Canonical Signaling |
|  | Lysophosphatidic Acid/LPARs Signaling |
|  | Ras-GRF Regulation Signaling |
|  | MERTK Signaling |
|  | Ras Signaling |
|  | AMPK Signaling |
|  | AXL Receptor Tyrosine Kinase Signaling |
|  | VEGF Signaling |
|  | Estrogens/ESR1 non-Genomic Signaling |
|  | EGFR Signaling |
|  | NF-kB Canonical Signaling |
|  | ERK/MAPK Canonical Signaling |
|  | TCR -> CREB/CREBBP/ATF Expression Targets |
|  | TCR -> NF-kB Expression Targets |
|  | TCR -> NFAT Expression Targets |
|  | TCR -> AP-1 Expression Targets |
|  | T-Cell Receptor -> NF-kB Signaling |
|  | T-Cell Receptor -> NFATC Signaling |
|  | T-Cell Receptor -> CREBBP Signaling |
|  | TGFA -> TP53 Expression Targets |
|  | TGFA -> HIF1A Expression Targets |
|  | TGFA -> MEF/MYOD/NFATC Expression Targets |
|  | TGFA/AP-1/ATF Expression Targets |
|  | TGFA -> CREB/CREBBP/ELK-SRF/MYC Expression Targets |
|  | Thrombopoietin -> AP-1/CREB/CREBBP/MYC Expression Targets |
|  | IL1B -> PGE2 Expression Targets |
|  | ROS in Neutrophil Mediated Cell Damage |
|  | ROS in Triggering Vascular Inflammation |
|  | Glutamate Mediated Neurotoxicity |
|  | Mitochondrial Damage Induced by Hypoxia |
|  | ROS and RNS in Vasoconstriction and Vasodilation Regulation |
|  | Ca2+ Cytosolic Overload |
|  | ROS in Angiotensin Mediated Cardiovascular Remodeling and Hypertrophy |
|  | PLAU -> ELK-SRF/AP-1 Expression Targets |
|  | VEGFA -> ATF/CREB/ELK-SRF Expression Targets |
|  | VEGFA -> NFATC Expression Targets |
|  | VEGFC -> ATF Expression Target |
|  | VEGFA -> AP-1/CREBBP/MYC Expression Targets |
|  | VEGFA -> NCOR2 Expression Target |
|  | VEGFR -> NFATC Signaling |
|  | VEGFR -> AP-1/CREB/MYC Signaling |
|  | VEGFR -> ATF/CREB/ELK-SRF Signaling |
|  | pathway 3 |
|  | e |
|  | pathway 4 |
|  | ace2 |
|  | New Pathway (2) |
|  | sialic |
|  | cypa |
|  | Model of PPARG signaling in psoriasis |
|  | Model of PPARG related pathways in psoriasis (short version) |
|  | New Pathway |
|  | hemophylia |
|  | dsRNA induces innate immunity and INFs in alveolar cells |
|  | SARS induces Complement Activation |
|  | Dendritic Cell Activation in COVID19 |
|  | 2\_Acute phase initiation in atopic dermatitis |
|  | 2\_Harlequin ichthyosis overview |
|  | 1\_The onset of atopic dermatitis |
|  | 1\_Osteoclast-mediated bone resorption in postmenopausal women |
|  | 2\_Osteoblast-mediated bone demineralization |
|  | 2\_Chondrocyte death in osteoarthritis |
|  | 1\_Formation of cysts in polycystic kidney disease |
|  | 2\_Dysfunction of cilia in polycystic kidney disease |
|  | 2\_Podocyte dysfunction in glomerulonephritis |
|  | 3\_Enhanced angiogenesis in endometriosis |
|  | 1\_Disruption of luteinizing hormone and follicle-stimulating hormone secretion in PCOS |
|  | 2\_Oxidative stress, all-trans-retinal, and lipofuscin toxicity in agerelated macular degeneration |
|  | 1\_Age-related cataract |
|  | 3\_Impairment of mechanoelectrical transduction and potassium cycling in the inner ear in hearing loss |
|  | 1\_Dysfunction of cochlear hair cell stereocilia proteins in hearing loss |
|  | 2\_Dysfunctions of cochlear hair cell synapse proteins in hearing loss |
|  | 2\_Pathogens stimulate mucins expression in the middle ear |
|  | 3\_Arterial wall inflammation and calcification in atherosclerosis |
|  | 2\_Oxidized LDL causes endothelial dysfunction in atherosclerosis |
|  | 1\_Endothelial cells stimulate vasoconstriction and thrombosis in pulmonary hypertension |
|  | 2\_Cardiomyocyte hypertrophy |
|  | 1\_Sarcomere disorganization and intracellular calcium overload |
|  | 3\_Cardiomyocyte dysfunction unrelated to cell junctions in ARVD |
|  | 3\_Dysfunction of smooth muscle cells in hypertension |
|  | 4\_Endothelial cells repress vasoconstriction in hypertension |
|  | 1\_Rise in aldosterone synthesis in adrenal zona glomerulosa cells |
|  | 2\_The intense proliferation and contractile activity of smooth muscle cells in pulmonary hypertension |
|  | 1\_Cigarette smoke causes dysfunction and production of inflammatory mediators in alveolar epithelial cells |
|  | 3\_1\_Mucus accumulation in asthma |
|  | 2\_1\_Eosinophilia and neutrophilia in asthma: eosinophil migration and activation |
|  | 2\_2\_Eosinophilia and neutrophilia in asthma: eosinophil apoptosis |
|  | 4\_1\_Airway smooth muscle cells contraction |
|  | 4\_2\_Airway smooth muscle cells proliferation and airway tissue remodeling |
|  | 4\_Mucin hyperproduction in goblet and mucous cells in COPD |
|  | 1\_CD4+ T cell and epithelial cell response in celiac disease |
|  | 2\_CD8+ T-cell response and mucosal damage in celiac disease |
|  | 5\_Tax-mediated dysregulation of NF-kB signaling in HTLV-1 infection |
|  | 1\_2\_Feedback relations between coagulation and fibrinolysis |
|  | 1\_1\_Dysfunction of coagulation cascade in hemophilia |
|  | 2\_Hyperglycemia and hyperlipidemia trigger beta-cell apoptosis |
|  | 3\_1\_Beta-cell proliferation and compensation: proliferation |
|  | 1\_2\_Insulin synthesis and secretion: insulin secretion |
|  | 1\_1\_Insulin synthesis and secretion: insulin processing and storage |
|  | 3\_2\_Beta-cell proliferation and compensation: neogenesis/dedifferentiation |
|  | 1\_CFTR expression and degradation in epithelial cells in cystic fibrosis (class I-II mutations) |
|  | 3\_Islet beta-cell destruction in T1D |
|  | 3\_Pain perception in diabetic neuropathy |
|  | 2\_Neuron and Schwann cell death |
|  | 1\_Glutamate and calcium excitotoxicity of motor neurons in ALS |
|  | 2\_Glutamate overload caused by mutations |
|  | 1\_1\_Glutamate overload enforced prostaglandins and NO synthesis in neurons |
|  | 1\_2\_Glutamate overload and NO-mediated vasodilation |
|  | 1\_Decreased secretion of thyroid hormones |
|  | 2\_1\_General pathways of thyroid hormone signaling |
|  | 2\_2\_Effects of thyroid hormone deficiency: cell-specific effects of thyroid hormone action |
|  | 1\_ab\_Amyloid-beta in Alzheimer's disease |
|  | 1\_cd\_Amyloid-beta in Alzheimer's disease |

---

|  |  |
| --- | --- |
| MedScan ID | 1093823 |
|  | 1284785 |

---

|  |  |
| --- | --- |
| Alias | Ca(+) |
|  | Ca ions |
|  | calcium cation |
|  | calcium (2+) |
|  | calcium(I) cation |
|  | Ca2+ ions |
|  | Ca2+ ion |
|  | Ca++ cation |
|  | Ca2(+) |
|  | calcium ion (1+) |
|  | calcium (II) ion |
|  | Ca cation |
|  | Ca++ cations |
|  | Ca++ ions |
|  | Ca 2+ |
|  | calcium(2+) ion |
|  | Ca2+ cations |
|  | Ca ion |
|  | Ca++ ion |
|  | calcium dication |
|  | calcium ion |
|  | calcium |
|  | calcium cations |
|  | calcium ions |
|  | Ca2+ cation |
|  | calcium(1+) ion |
|  | Ca cations |
|  | calcium(1+) |
|  | Ca2+ |
|  | Ca(2+) |
|  | calcium monocation |
|  | calcium ++ |
|  | calcium (II) ions |
|  | Ca++ |
|  | 14102-48-8 |
|  | calcium(2+) ions |
|  | 14127-61-8 |

---

|  |  |
| --- | --- |
| CAS ID | 14127-61-8 |
|  | 14102-48-8 |

---

|  |  |
| --- | --- |
| ChEBI ID | 29108 |
|  | 39099 |

---

|  |  |
| --- | --- |
| HMDB ID | HMDB00464 |

---

|  |  |
| --- | --- |
| KEGG ID | C00076 |

---

|  |  |
| --- | --- |
| InChIKey | BHPQYMZQTOCNFJ-UHFFFAOYSA-N |

---

|  |  |
| --- | --- |
| Molecular Formula | Ca+2 |

---

|  |  |
| --- | --- |
| PubChem SID | 135062483 |

---

|  |  |
| --- | --- |
| PubChem CID | 271 |

---

|  |  |
| --- | --- |
| IUPAC Name | calcium(2+) |

---
